# Magnetic resonance imaging-based cerebral tissue classification reveals distinct spatiotemporal patterns of changes after stroke in non-human primates

**DOI:** 10.1186/s12868-015-0226-7

**Published:** 2015-12-15

**Authors:** Mark. J. R. J. Bouts, Susan. V. Westmoreland, Alex J. de Crespigny, Yutong Liu, Mark Vangel, Rick M. Dijkhuizen, Ona Wu, Helen E. D’Arceuil

**Affiliations:** Department of Radiology, Athinoula A. Martinos Center for Biomedical Imaging, Massachusetts General Hospital, 149 13th Street CNY 2301, Charlestown, MA 02129 USA; Biomedical MR Imaging and Spectroscopy Group, Image Sciences Institute, University Medical Center Utrecht, Utrecht, The Netherlands; New England Primate Research Center, Pathology, Southborough, MA USA; Department of Radiology, The University of Nebraska Medical Center, Omaha, NE USA

**Keywords:** ISODATA, Tissue signatures, Non-human primates, Stroke, Temporal ischemic tissue evolution, Diffusion-tensor imaging

## Abstract

**Background:**

Spatial and temporal changes in brain tissue after acute ischemic stroke are still poorly understood. Aims of this study were three-fold: (1) to determine unique temporal magnetic resonance imaging (MRI) patterns at the acute, subacute and chronic stages after stroke in macaques by combining quantitative T_2_ and diffusion MRI indices into MRI ‘tissue signatures’, (2) to evaluate temporal differences in these signatures between transient (n = 2) and permanent (n = 2) middle cerebral artery occlusion, and (3) to correlate histopathology findings in the chronic stroke period to the acute and subacute MRI derived tissue signatures.

**Results:**

An improved iterative self-organizing data analysis algorithm was used to combine T_2_, apparent diffusion coefficient (ADC), and fractional anisotropy (FA) maps across seven successive timepoints (1, 2, 3, 24, 72, 144, 240 h) which revealed five temporal MRI signatures, that were different from the normal tissue pattern (P < 0.001). The distribution of signatures between brains with permanent and transient occlusions varied significantly between groups (P < 0.001). Qualitative comparisons with histopathology revealed that these signatures represented regions with different histopathology. Two signatures identified areas of progressive injury marked by severe necrosis and the presence of gitter cells. Another signature identified less severe but pronounced neuronal and axonal degeneration, while the other signatures depicted tissue remodeling with vascular proliferation and astrogliosis.

**Conclusion:**

These exploratory results demonstrate the potential of temporally and spatially combined voxel-based methods to generate tissue signatures that may correlate with distinct histopathological features. The identification of distinct ischemic MRI signatures associated with specific tissue fates may further aid in assessing and monitoring the efficacy of novel pharmaceutical treatments for stroke in a pre-clinical and clinical setting.

## Background

Diffusion-weighted imaging (DWI) is sensitive for the early detection of ischemic tissue injury due to stroke, with changes being visible as early as 11 min post-onset [1]. Greater understanding of the temporal evolution of DWI under conditions of ischemia and revascularization is important for accurate diagnosis and management of stroke patients. Serial MRI studies performed in experimental rat stroke models have shown that the spatiotemporal progression of tissue injury after focal ischemia is heterogeneous and differs between animals that revascularize and those that do not [2]. However, because rodents have intrinsically different stroke evolution patterns than primates [3], serial studies using gyrencephalic primates may potentially provide more representative insight into human stroke [4]. A previous study on post-stroke changes in T_2_ and diffusion tensor imaging (DTI) found that lesion evolution after permanent and transient stroke in cynomologous macaques is more consistent with changes observed in human stroke patients, compared to rodent stroke models [5]. In that study, differences in lesion evolution were investigated using volumetric analysis that may introduce bias from regional tissue averaging, potentially obscuring local temporal variations in lesion development [6, 7].

Several studies have demonstrated that voxel-based cluster analysis can provide improved insights into the local characteristics of regional tissue changes after stroke [6–8]. Iterative self-organizing data analysis (ISODATA) circumvents the need for substantial user-interaction and a priori specification of the number of clusters by dynamically determining the optimal number of clusters [6, 7]. ISODATA has been used to create tissue signatures that correlated with lesions on radiological outcomes in human stroke and to histological outcome in lissencephalic rodent stroke models [2, 8, 9]. Yet, the utility of ISODATA for investigating gyrencephalic non-human primate stroke models for which histological tissue outcome can confirm tissue injury or recovery remains unexamined. This study, therefore, investigated the evolutionary changes of diffusion and T_2_ MRI indices after stroke in cynomologous macaques using a modified ISODATA analysis. The first aim of this study was to identify unique temporal MRI patterns in the acute, subacute, and chronic stages after stroke by combining multiple MRI measures on a voxel-wise basis. The second aim focused on characterizing spatial and temporal differences in T_2_ and DTI parameters between macaques with transient versus permanent middle cerebral artery occlusion (MCAo). The final aim of this study was to compare the MRI-determined tissue signatures with histopathology.

## Results

### Stroke model

Analysis was conducted on two macaques with permanent MCAo (pMCAo1, pMCAo2) and two macaques with 3-h transient MCAo (tMCAo1, tMCAo2). For three animals (two permanent and one 3-h transient MCAo), spatially and temporally adjusted ISODATA (ST-ISODATA) was used to combine serial DTI and T_2_ data from approximately 1 h (1.33 ± 0.30 h), 2 h (2.41 ± 0.44 h), 3 h (3.04 ± 0.44 h), 24 h (23.34 ± 1.57 h), 72 h (70.26 ± 1.54 h), 144 h (142.18 ± 1.83 h), and 240 h (232.22 ± 9.92 h) after stroke onset. For the fourth animal (tMCAo2) ISODATA cluster analysis included MRI up till 144 h, since T_2_ maps at 240 h were unavailable. For “chronic” lesion volumes, the 17-day (406.98 ± 2.31 h) T_2_ maps were used except for one animal (pMCAo2) that died before the 17-day time-point; in that case the 10 days data was used instead.

### Tissue classifications

In the permanent MCAo group, 48 % of the voxels with abnormal tissue signature were assigned to the *Core* tissue class. 41 % of the voxels were initially normal but became abnormal by 17 days (*Growth*). *Recovery* and *Edema* areas comprised 4 and 7 % respectively of the abnormal signature voxels. For the transient MCAo group, the majority of voxels were assigned to the *Edema* tissue class (58 %, P = 0.002), while the *Core* tissue class was smaller (14 %, P = 0.07). Percentage-wise, there were more *Growth* voxels in the permanent (41 %) than in the transient MCAo group (14 %) (P = 0.10), whereas *Recovery* voxels made up a larger percentage of the transient MCAo group (14 %) (P = 0.02).

### ISODATA-based tissue signatures

**Fig. 1:**
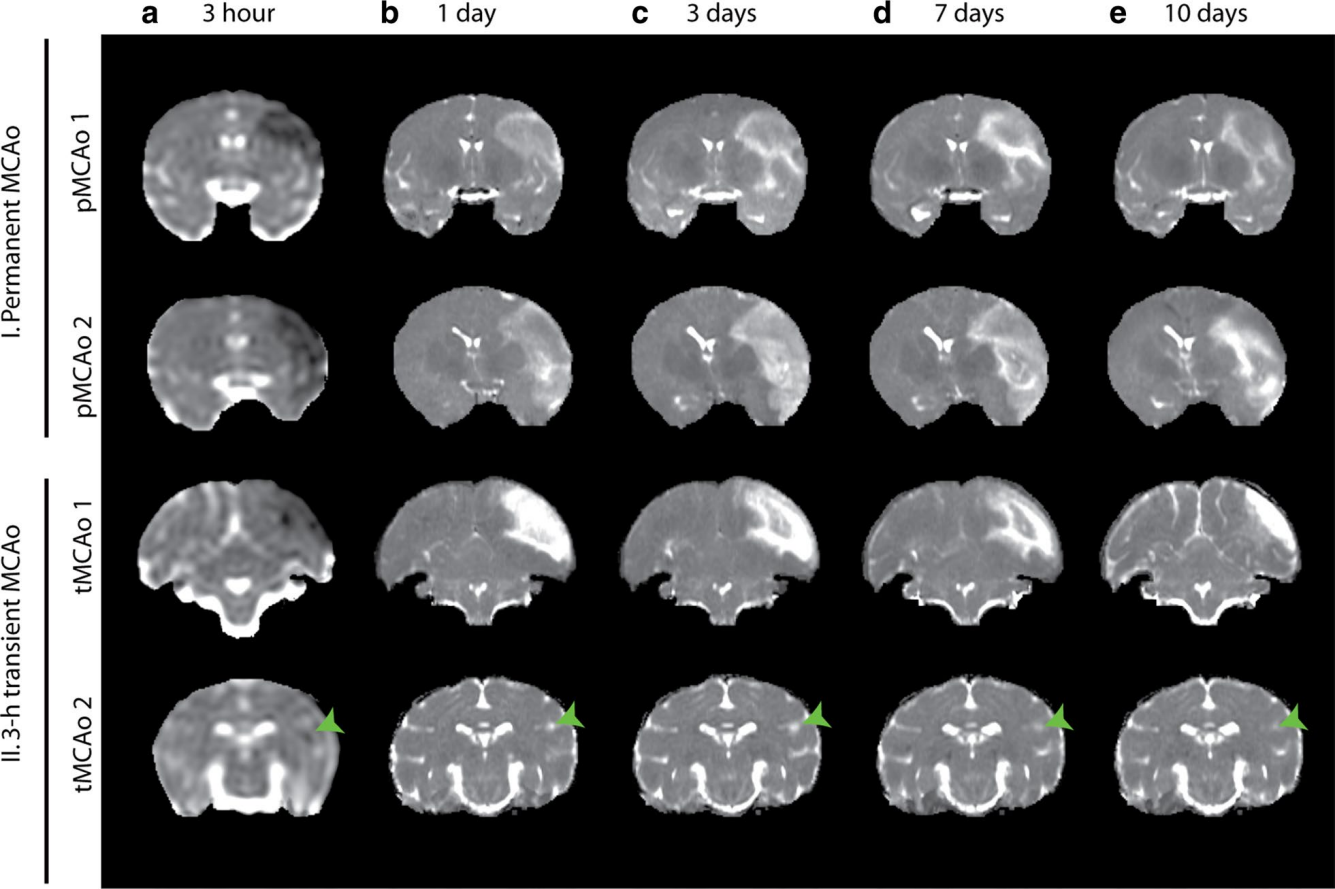
Tissue lesion development over five different timepoints in the four macaque brains. Coronal slices of the brains of permanent (I) and 3 h transient MCAo macaques (II). 3 h post onset ADC (**a**) maps are shown followed by 1 (**b**), 3 (**c**), 7 (**d**), and 10 (**e**) day T_2_ maps illustrating tissue lesion progression over time (10 day T_2_ map of M303 was unavailable, 17 day T_2_ map is shown instead; *green arrow* heads indicate the lesion for M303)

**Fig. 2:**
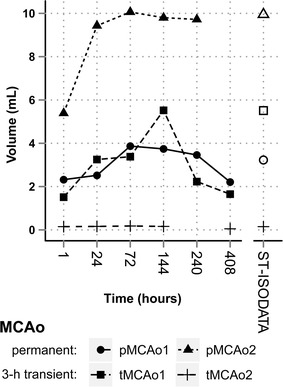
Temporal change in abnormal tissue lesion volumes compared with ST-ISODATA calculated abnormal tissue signature volumes. Abnormal tissue lesion volumes (whole markers) and ST-ISODATA assigned abnormal tissue signature volumes (open markers) in the four macaques. Largest tissue lesion volumes represented regions of ‘*Maximal Lesion*’ and were used for parameter optimization of ST-ISODATA. pMCAo2 died before 408 h follow-up, the 240 h was used as follow-up instead. tMCAo2 240 h T_2_ was not available

**Fig. 3:**
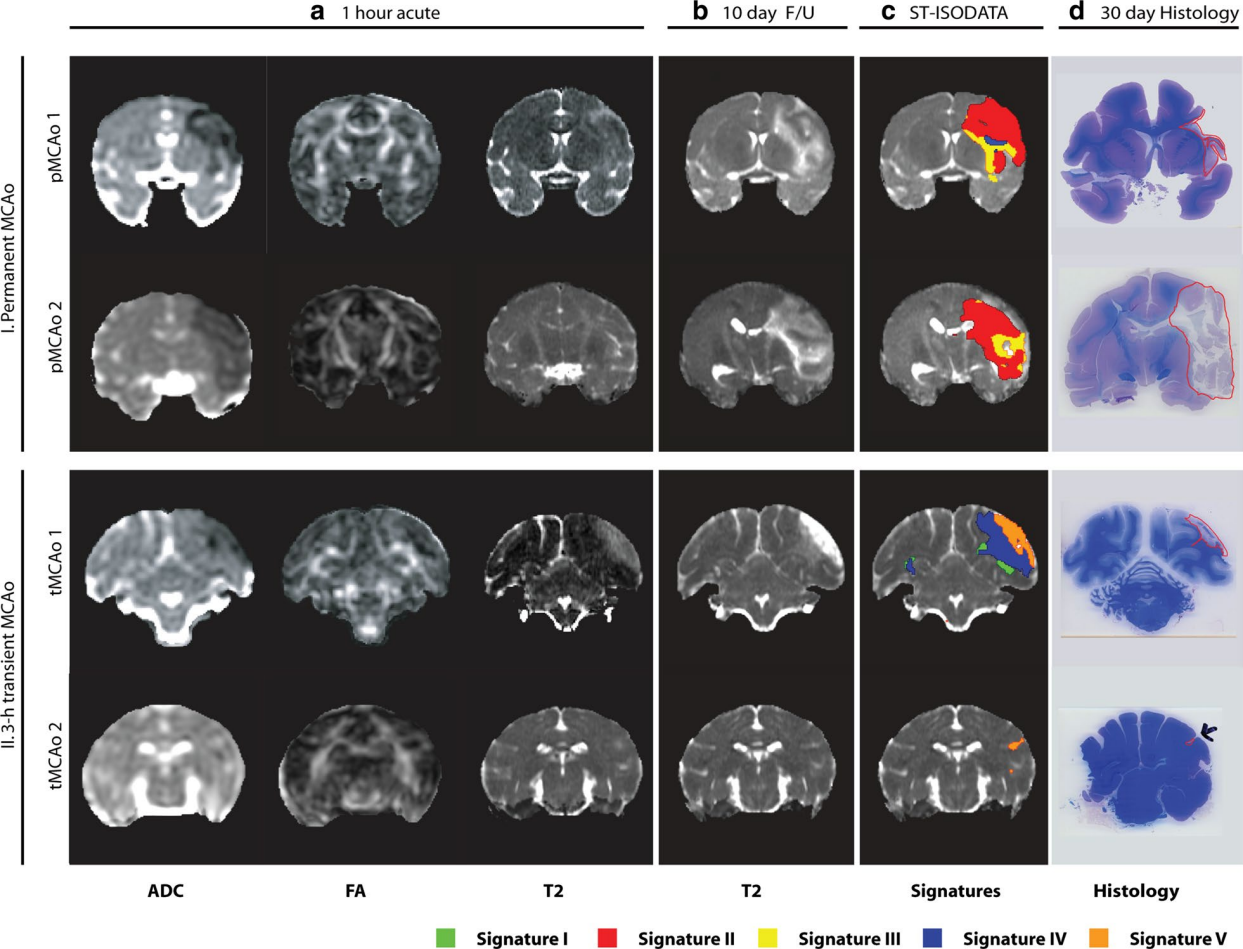
Example of coronal brain slices from macaques after permanent or 3 h transient MCAo. Quantitative ADC, FA, and T_2_ maps at 1 h after stroke induction (**a**), and 240 h follow-up T_2_ maps (**b**) of animals with permanent (I) or 3 h transient MCAo (II) are shown. For each animal the quantitative maps from 1 h up to 240 h after MCAo were combined using a ST-ISODATA approach resulting in five abnormal signatures (**c**; overlaid on 240 h follow-up T_2_ maps), that were consistent with regions of affected tissue on LFB-stained histological sections (*red-outlined regions*; **d**)

Figure 1 shows examples of tissue lesions as measured with ADC and T_2_ across time for the four MCAo brains. Figure 2 shows the measured lesion volumes across time from all brains. Overlap (i.e. Dice Similarity Index DSI) between the spatially and temporally adjusted ISODATA (ST-ISODATA) identified lesions and ‘*Maximal Lesion’* ROI was significantly different depending on the input MRI-parameters used. Highest DSI was obtained with a combination of ADC, FA, and T_2_ (DSI = 0.80 ± 0.17) (Table 1). Therefore combined ADC, FA, and T_2_-based ST-ISODATA was subsequently used with the minimum number of voxels within a cluster (Φ_N_), the minimum inter-cluster distance (Φ_C_), and the maximum allowed intra-cluster dispersion (Φ_S_) set to 100, 0.98 ± 0.34, and 0.52 ± 0.03, respectively. This resulted in 12–17 clusters that were identified for each brain. Subsequent coefficient of variance (CoV) pruning with a threshold of 0.05, the threshold with highest overlap between ST-ISODATA identified lesions and ‘*Maximal Lesion’* ROI (DSI = 0.80 ± 0.16), reduced the number of identified clusters to 2–6 per brain. The resulting normalized ipsilateral clusters were pooled across brains, creating six signatures with ranges 1–5 (Signature N), 6–15 (Signature I), 26–35 (Signature II), 36–45 (Signature III), 46–55 (Signature IV), or 56–65 (Signature V). No cluster values ranging from 16 to 25 or 66 to 100 were observed and therefore signatures were not generated encompassing these cluster values. Signature N was consistent with unaffected tissue while the other five indicated varying degrees of tissue abnormality corresponding to histologically-identified affected tissue areas at 30 days. Figure 3 shows examples of acute (1 h) and 240 h MRI datasets (a–d) from brains with permanent MCAo (I: pMCAo1 and pMCAo2) and brains with 3-h transient MCAo (II: tMCAo1 and tMCAo2) along with resultant signatures.

**Table 1:** Overlap of abnormal ST-ISODATA tissue volumes and ‘*Maximal Lesion’* volumes measured using Dice’s Similarity Index

ST-ISODATA input MRI	DSI
ADC	0.58 ± 0.39*
FA	0.35 ± 0.24
T2	0.59 ± 0.29*
ADC, FA	0.68 ± 0.32**
ADC, T_2_	0.60 ± 0.41*
ADC, FA, T_2_	0.80 ± 0.16**

The combination of ADC, FA, and T_2_ as input for ST-ISODATA clustering resulted in highest overlap (i.e. highest Dice’s similarity index values) between ST-ISODATA calculated abnormal tissue volumes and ‘*Maximal Lesion*’ volumes. Therefore this combination was used for ST-ISODATA analysis

*DSI* Dice’s similarity index

* P < 0.05 versus FA, ** P < 0.001 versus FA

**Fig. 4:**
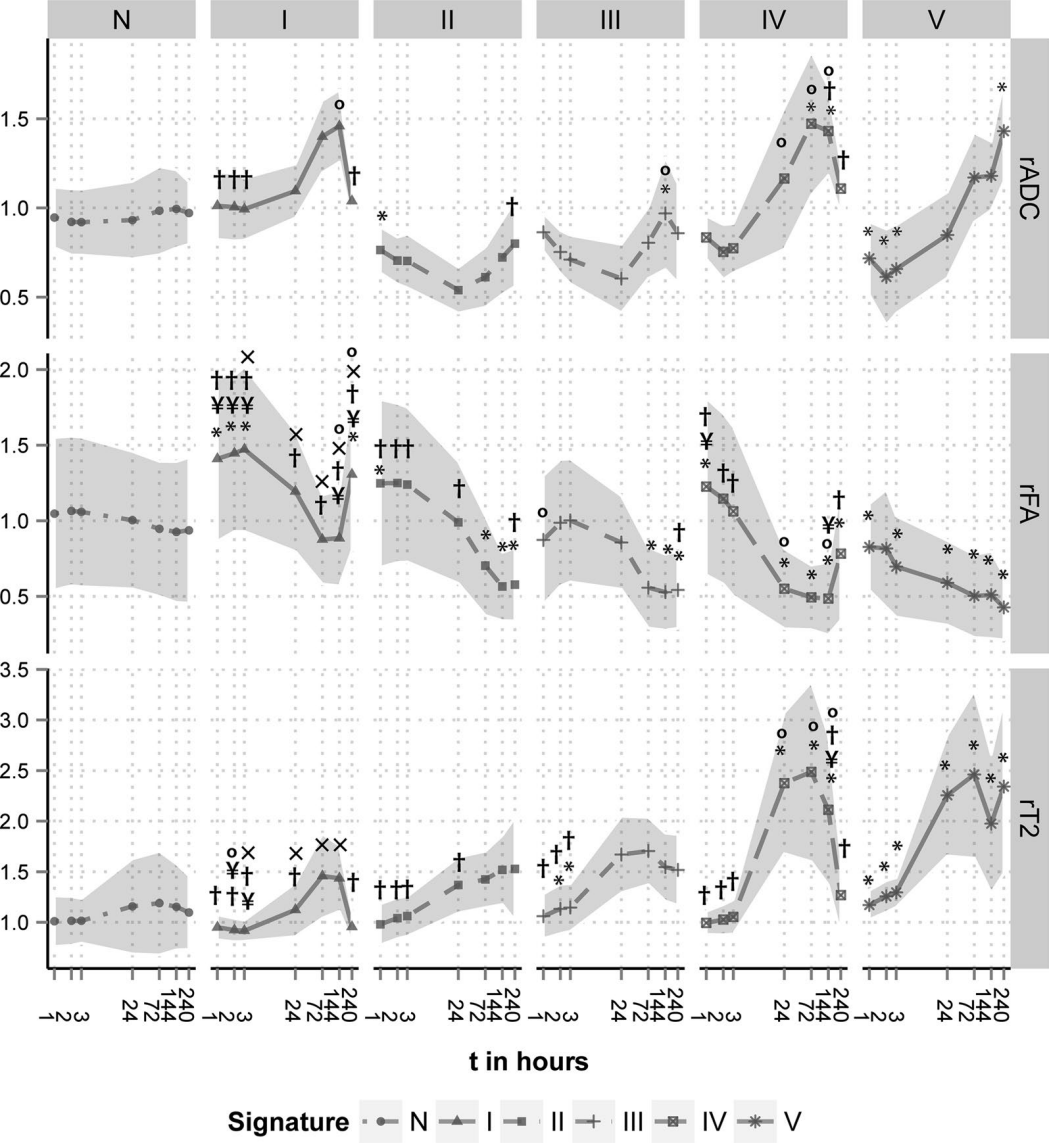
Temporal change in rADC, rFA, and rT_2_ of ST-ISODATA assigned tissue signatures. Plots of mean rADC (**a**), rFA (**b**), and rT_2_ (**c**) as a function of time after stroke induction. ST-ISODATA identified one normal (N) and five abnormal signatures (I–V) that evolved differently from each other (P < 0.0001). (*P < 0.05 versus Signature N, °P < 0.05 versus Signature II, ^¥^P < 0.05 versus Signature III, ^x^P < 0.05 versus Signature IV, ^†^P < 0.05 versus Signature V. *Shaded* *area* are standard deviations)

The five identified abnormal signatures showed differences in the temporal evolution of relative ADC (rADC), FA (rFA) and T_2_ (rT_2_) (P = 0.002) (Fig. 4). Decreased acute rADC were observed for Signature II, IV, and V (P < 0.001 versus Signature N), with largest rADC decrease for Signature II and particularly V (P < 0.03 versus all other Signatures). Decreased rADC were followed by a sharp increase in rADC in the first 24 h for Signature IV, whereas for Signature II rADC further decreased (P = 0.05). For Signatures II and III, rADC was reduced for at least 24 h before slowly increasing towards normal values (Signature II: P = 0.01 24 h versus 240 h; Signature III: P = 0.05 24 h versus 144 h). In the chronic phase, Signatures I and IV showed a slow decrease, whereas Signature V exhibited a progressive increase in rADC (P = 0.02 24 h versus 240 h). Decreased values in acute rFA were only observed for Signature V (P < 0.01 versus Signature N). All signatures showed a decreasing trend in rFA up until 144 h. After 144 h, rFA started to normalize in Signatures I and IV. For Signatures I and IV, rT_2_ increased until 144 h, after which a gradual decrease was observed. Signature III displayed progressive increase in rT_2_, until stabilizing at 72 h. The rate of increase in rT_2_ after 3 h was greater for Signature IV compared to the other signatures (P < 0.01), except for Signature V. For Signatures II and V, rT_2_ progressively increased across all time-points. However, rT_2_ values at 240 h differed statistically significantly from normal tissue only for Signature V (P = 0.003).

**Fig. 5:**
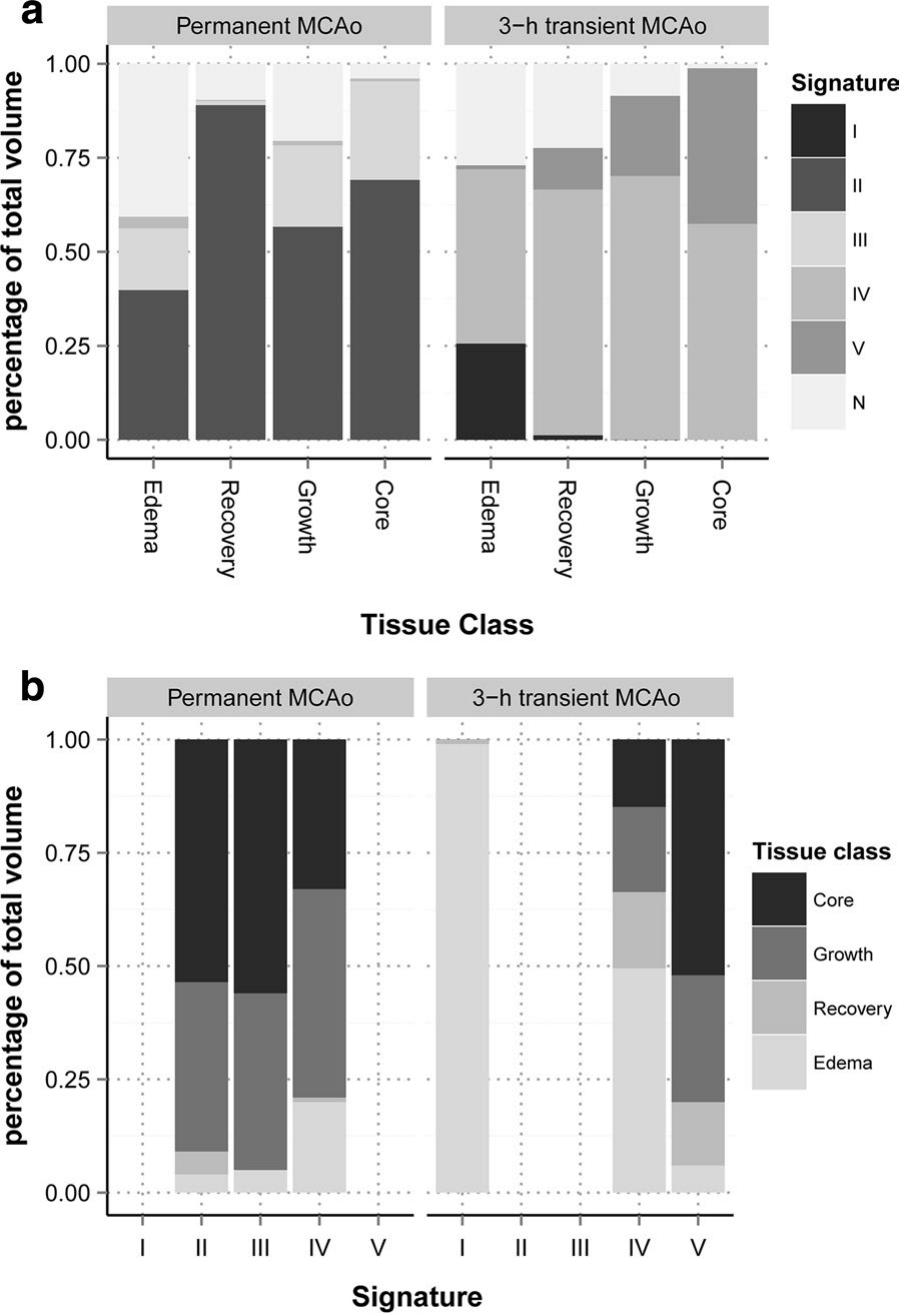
Temporal tissue signature distributions. Percentage distribution of the temporal tissue signatures over the four tissue classifications (**a**) and the four tissue classes over the five abnormal signatures (**b**). In the permanent MCAo group (N = 2), tissue classes consisted predominantly of Signatures II and III, particularly depicting *Core* and *Growth* types. Signature V was found only in the transient MCAo group (N = 2). In this group the most prevalent signature across all tissue classes was IV. The *Edema* type made up 99 % of Signature I and 46 % of Signature IV in the transient MCAo group, but only 20 % of Signature IV for the permanent group (P < 0.0001)

The distribution of signatures differed between permanent and transient MCAo groups (P < 0.0001). The permanent MCAo group was primarily composed of Signatures II and III while the transient MCAo group consisted mostly of Signatures IV and V. For both transient (P < 0.0001) and permanent MCAo (P = 0.006) groups, the distribution of the six ST-ISODATA signatures differed between the four tissue classes (*Core*, *Edema*, *Growth*, *Recovery*) (Fig. 5a). In one brain (tMCAo1), Signature I was found almost exclusively within areas of *Edema* (98.8 %), with some voxels in regions of *Growth* (0.1 %) and *Recovery* (1.1 %) (Fig. 5b). Signature IV was found in permanent but particularly in 3-h transient MCAo brains (P < 0.0001), in the latter predominantly in regions of *Edema* (49 %), and to lesser extent in regions of *Core* (15 %) (P = 0.02), *Growth* (19 %) (P = 0.05) and *Recovery* (17 %) (P = 0.03).

### Histological features

**Fig. 6:**
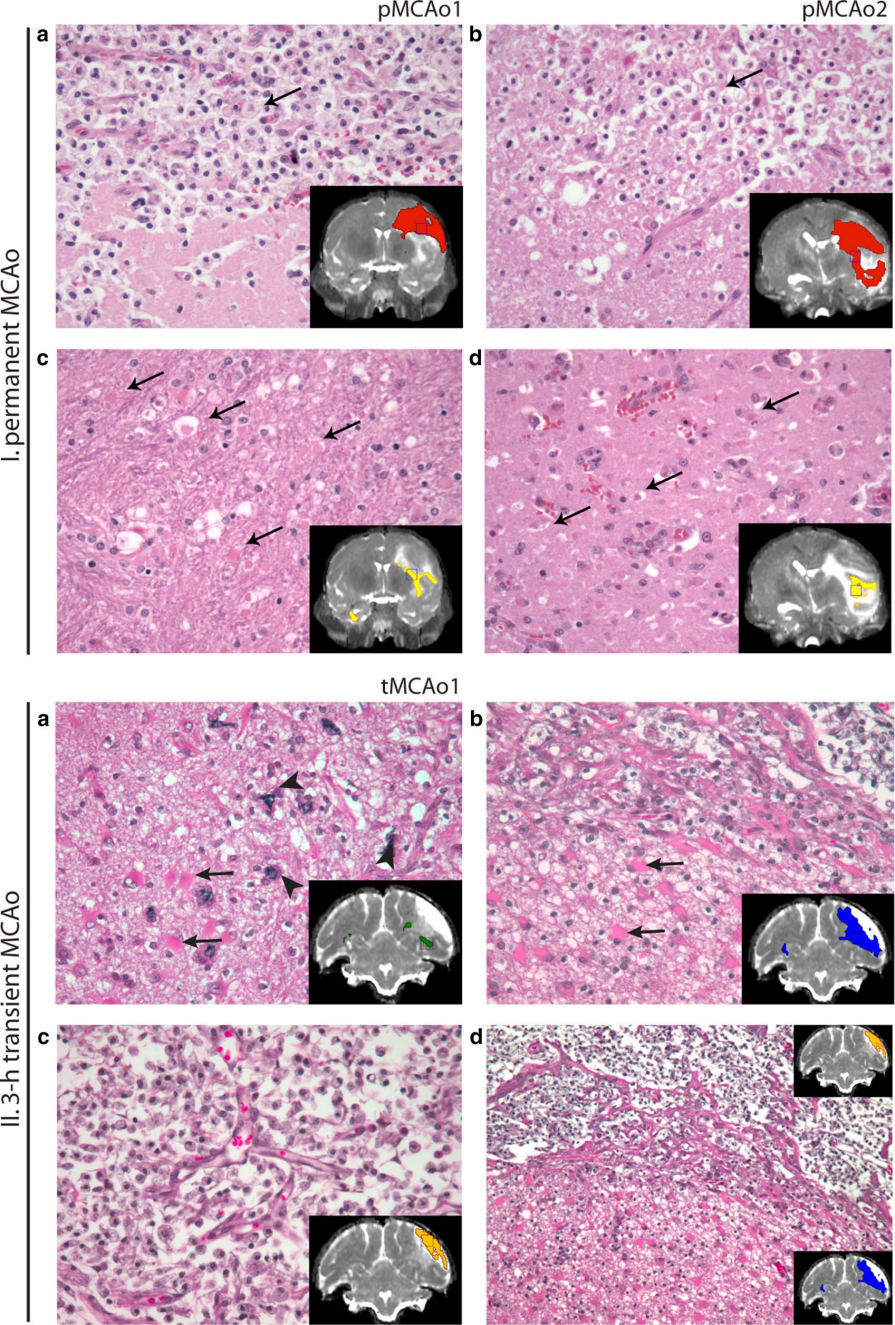
Comparison of ST-ISODATA designated abnormal tissue signatures with histology. H&E stained brain sections from animals with permanent (pMCAo1: I.**a**, **c** and pMCAo2: I.**b**, **d**) or 3-h transient (tMCAo1: II.**a**–**d**) MCAo. Insets display the 240 h T_2_ MRI with overlays of Signatures I (*green*), II (*red*), III (*yellow*), IV (*blue*), and V (*orange*), and a squared box indicating the area of the microscopic fields. In permanent MCAo, most severe tissue injury was observed in Signature II regions (I.**a**, **b**) that corresponded with severe tissue necrosis and loss, sheets of activated, vacuolated microglia/macrophages (gitter cells, *thin arrows*) and some remaining vasculature. Neuronal and glial injury and degeneration in the peri-lesional brain regions corresponded with Signature III, and included degenerated axons (spheroids, I.**c**, *thin arrows*) and hypereosinophilia of dying neurons (I.**d**, *thin arrows*). In transient MCAo Signature I (II.**a**) corresponded with areas with peri-lesional neuronal mineralization (*fat arrows*) and astrogliosis evidenced by the presence of numerous, large eosinophilic gemistocytes (*thin arrows*). Signature IV (II.**b**) corresponded with brain regions with vascular proliferation, spongiosis, and marked astrogliosis. Signature V (II.**c**) corresponded with brain regions with necrosis and infiltration of gitter cells. The border region between Signatures IV and V is displayed in lower magnification in II.D (×10). Original magnification ×20 (I.**a**–**d**; II.**a**–**c**)

Hematoxylin and eosin (H&E), luxol fast blue (LFB) and myelin basic protein (MBP)-stained brain sections from all cases were analyzed with particular attention given to regions identified by Signatures I through V. Lesion characteristics from each available signature area from the four brains are summarized in Table 2. Histopathological alterations in the brain included extensive necrosis with brain tissue loss and cavitation; axonal swelling; astrogliosis; edema and spongiosis; vascular proliferation; and the presence of activated and foamy microglia/macrophages (gitter cells) with phagocytized necrotic debris. In the permanent MCAo brains, Signature II was primarily found in gray matter (GM) areas (central, supra marginal and temporal gyri) and corresponded to the most severe lesions with areas of extensive necrosis, tissue loss, dead neurons, and gitter cells (Fig. 6I). Signature III was detected solely in permanent MCAo brains in areas of white matter (WM) (external capsule, and precentral and temporal WM) and gray-white matter junctions (parietal and temporal cortices). These regions showed areas of neuronal injury with features of degenerating neurons and axons, but absence of gitter cells. Parietal temporal WM was also affected in Signatures IV and V defined volumes. Signature IV corresponded to a prominent border region between infarcted and surviving tissue with vascular proliferation, spongiosis, and astrogliosis. These histological changes were found in both permanent and transient MCAo brains. Astrogliosis along with some neuronal mineralization and edema were also observed in Signature I, present in only one transient MCAo brain (Fig. 6II).

**Table 2:** Histological characteristics of the ST-ISODATA abnormal tissue signatures within the brains of the four macaques

MCAo	Signature				
	I	II	III	IV	V
Permanent					
pMCAo1	NA	Marked necrosis with dead neurons, tissue loss with gitter cells	Degenerate neurons, degenerate axons (spheroids)	Spongiosis, vacuolation, astrogliosis, vascular proliferation	NA
pMCAo2	NA	Marked necrosis with dead neurons, tissue loss with gitter cells	Degenerate neurons, degenerate axons (spheroids)	NA	NA
3-h transient					
tMCAo1	Edema, spongiosis, astrocytosis, mineralization	NA	NA	Spongiosis, vacuolation, astrogliosis, vascular proliferation	Edema, necrosis, gitter cells
tMCAo2	NA	NA	NA	NA	Edema, necrosis, gitter cells

*NA* not applicable

## Discussion

In this study, T_2_ and diffusion (ADC, FA) MRI indices acquired at acute, subacute, and chronic stages after stroke were combined on a voxel-wise basis to identify unique spatiotemporal MRI profiles, and to determine differences in stroke evolution after permanent or transient MCAo in non-human primates. The ST-ISODATA approach identified six distinct tissue signatures, with one signature consistent with non-ischemic normal tissue. The five other (abnormal) signatures showed substantial differences from this normal signature. These abnormal signatures exhibited differences in tissue class distribution and temporal profile depending on duration of occlusion (permanent or transient) and level of neuronal damage.

Despite its general use, the utility of single acute MRI parameters like T_2_ or ADC for the prediction of tissue infarction are still being debated [10–13]. As we have demonstrated, using a single MRI parameter such as ADC cannot reliably identify irreversibly damaged “core” tissue, since tissue with decreased ADC may “recover” at subsequent timepoints as a result of early reperfusion. The merits of using multiparametric MRI measures on a voxel-wise basis have been previously illustrated in rodent [2, 7, 9] and in human studies [14, 15]. Yet, previous histological validation of multiparametric MRI-based algorithms was primarily based on lissencephalic rodent models [2, 9, 16] for which translation of findings to human subjects are not straightforward [4]. Therefore recent guidelines recommend further validation using higher order animals, e.g. non-human primates as employed in this study, to improve insights based on increased anatomic similarity with human brains [4].

Variations in temporal evolution of signatures classified as abnormal may inform on varying degrees of neuronal damage [5, 10, 17–20]. Increased or decreased acute FA values may be associated with different levels of cell swelling in white or gray matter structures [17, 19, 21]. Alternatively, these FA alterations may reflect initially preserved cytoarchitecture or increased extracellular space tortuosity as a result of cytotoxic edema (observed as a reduction of rADC) that gradually decayed as ischemia progressed towards tissue necrosis (manifested as increases in rADC) [19]. Regions of relatively acutely increased FA were observed in areas of cortical gray matter while decreased FA were primarily observed in temporal and parietal white matter regions. Future studies should extensively examine differences in gray and white matter FA evolution after ischemia. The differences in ADC pseudo-normalization and increased T_2_ may have been accelerated by reperfusion. Reperfusion may reinstate energy metabolism and ion-pump function effecting permanent or transient reversal of ADC [12, 22, 23]. Alternatively flow restoration may aggravate blood–brain-barrier disruption resulting in vasogenic edema [17, 24]. Subsequent renormalization of T_2_ (along with ADC and FA) in the more chronic stages may most likely be associated with resolution of edema [25]. However, it may also highlight areas with delayed tissue damage [18] or more likely (in our case) long-term tissue remodeling. Regions that exhibited increased FA in chronic stages were also associated with glial scar tissue. At early time-points, these same regions typically showed reduced FA values, most likely as a result of early edema from reperfusion. As edema resolved, these reduced FA values renormalized, ultimately increasing to values above normal tissue, consistent with findings reported in chronic rodent stroke models [26–28].

Cluster analysis may be affected by the quality of the underlying data. Biologically induced deformations (like edema) or imaging induced distortions (like residual eddy current distortions or susceptibility artifacts) may contribute to an increased number of (artefactual) clusters that may negatively influence subsequent analysis [29]. In this study several pruning steps—including spatial contiguity constraints, CoV analysis, and advanced (non-) linear registration—were employed to reduce these potential artifacts. However, despite these efforts not all artifacts could be resolved and therefore signature analysis was restricted to the ipsilateral parenchyma only. It may be furthermore argued that CoV pruning and the “intra-dataset” signature merging steps may oversimplify or obscure additional tissue profiles. Nevertheless, we identified biologically interpretable MRI signatures that corresponded with specific features of tissue damage as verified with histology and showed highest overlap between ISODATA-segmented abnormal tissue regions and the manually-defined ‘*Maximal Lesion*’ regions. Our study was furthermore limited by retrospective analysis of a small cohort of animals precluding generalization of specific MRI tissue signatures heralding histological tissue damage. However, the scope of the current retrospective study is to analyze the MRI data with regard to size and characterization of lesions. Future prospective studies should investigate histopathological changes in individual brains to assess the predictive value of these MRI tissue signatures for infarct evolution and to compare these changes with pre-specified cellular markers (e.g. microglia, macrophages, leukocytes, astrocytes, blood vessels) that could further inform about the potential origin of the observed diverse MRI signals. Abnormal cluster regions from brains with relatively large lesions (like tMCAo1, pMCAo1) corresponded better with ‘*Maximal Lesion’* regions than those with smaller lesions (like tMCAo2) and possibly dominated clustering results. Subtle temporal changes that may occur with smaller tissue lesions may as a result be obscured. Another limitation is the relatively large field-of-view and low spatial-resolution that was used as a result of performing these studies on clinical large bore scanners. Future studies using MRI systems with higher gradient strengths and specialized head coils would allow for acquisitions with higher spatial resolution [30]. Diffusion measures, and hence cluster results, may also be improved with additional number of gradient directions. In the future, multiparametric MRI analysis should be validated on larger cohorts of animals to establish robust categories of tissue signatures. This may open opportunities for improved insights regarding treatment-induced changes and provide an additional tool for optimizing the translation of experimentally obtained findings to human stroke applications.

## Conclusions

This study highlighted the applicability and potential of multiparametric MRI-based algorithms to identify ischemic tissue sub-types and to further characterize tissue progression after stroke in gyrencephalic brains. Despite the limited number of animals included in this study, the spatio-temporal assessment of MRI parameters enabled us to distinguish distinct tissue evolution patterns that represented specific histological outcome.

## Methods

### Acute stroke model

This study involved a retrospective analysis of data acquired previously as part of a study for which all procedures were approved by the Subcommittee for Research Animal Care (SRAC), the Institutional Animal Care and Use Committee (IACUC) of our institution that follows the National Institutes of Health Guide for the Care and Use of Laboratory Animals. Unilateral stroke was induced in seven adult male macaques (Macacca fascicularis, 7.7 ± 1.2 kg, 6–12 years old) by obstruction of the M1 branch of the middle cerebral artery (MCA) either by injection of a small volume of cyanoacrylate creating a permanent MCAo (n = 2), or by transient insertion of a micro infusion catheter. The catheter was subsequently removed after 3 h to effect reperfusion for a transient MCAo model (n = 5). Stroke induction followed procedures previously described [31]. Briefly, animals were sedated with diazepam (1 ml), then anesthesia was induced with atropine (0.04 mg/kg, i.m.) and ketamine (10 mg/kg, i.m.) and maintained either with isoflurane (2–3 %) in a 80/20 air/oxygen mixture or with propofol (300 µg/kg per hour, i.v.) in combination with remifentanil (0.1 µg/kg per hour, i.v.). Animals were mechanically ventilated with 20 % oxygen in air mixture to maintain end-tidal CO_2_ between 30 and 40 mm Hg, and physiological signs were monitored continuously.

Three 3-h transient MCAo group animals were excluded from analysis because of incomplete occlusion (N = 1), extensive imaging artifacts (N = 1), and MRI scanner problems in the hyperacute stage after stroke onset (N = 1).

### MRI

Serial MRI data were acquired on a 1.5 T MRI scanner (GE Signa) at 1 h intervals up to 6 h post-MCAo, and at 1, 3, 7, 10, 17, and 30 days. DTI (monopolar single-shot echo planar imaging, repetition time (TR)/echo time (TE) 8400/65.9 ms, 128 × 128 matrix, number of scans (NEX) 1, b-values of 0 (b_0_) and 1000 s/mm^2^ (6 directions), field-of-view (FOV) 200 mm, 3 mm slice thickness), and dual echo T_2_-weighted MRI (fast spin echo, TR/TE1/TE2 4200/10.7/95.9 ms, echo train length 16, 512 × 512, NEX 2, FOV 200 mm, 2 mm slice thickness, 0.5 mm slice gap) were obtained. The apparent diffusion coefficient (ADC) and fractional anisotropy (FA) maps were calculated from eddy-current corrected DTI datasets (MRVision, Winchester, MA, USA); T_2_ maps were calculated from the T_2_-weighted images.

### Data analysis

#### Pre-processing

Serial T_2_-maps and DTI were spatially aligned to the 1-h post MCAo b_0_ image using a two-step co-registration procedure. In the first step global alignment was achieved using a full affine, mutual information based registration procedure (Montreal Neurological Institute (MNI), Autoreg) [32]. In the second step, ventricular distortions, mid-line shifts, and sequence-induced distortions were largely compensated for using a mutual information based B-splines approach with a specialized cost function that enforced rigid transformations for particular volumes of interest (VOI) [33]. This prevented local lesion volume expansion or compression, effectively reducing potential co-registration induced artifacts.

Before cluster analysis, surrounding muscle and skull tissue was removed from the MRI (FMRIB Software Library (FSL) Brain Extraction Tool) [34]. Tissue with ADC values greater than 1.2 × 10^−3^ mm^2^/s at the first time-point, mostly arising from CSF and large vessels, were excluded [2]. The resulting segmentation served as a mask for the cluster analysis. Relative ADC (rADC), FA (rFA) and T_2_ (rT_2_) maps were calculated by dividing each map by its mean contralateral hemispheric value.

#### ISODATA cluster analysis

Temporal lesion evolution was assessed using a spatially and temporally adjusted ISODATA (ST-ISODATA) approach [6, 7]. ST-ISODATA determined the number of clusters (K) based on the underlying data rather than by prior specification of the expected number of clusters. A detailed description of the original algorithm can be found elsewhere [2, 9]. In brief, ISODATA is a K-means related unsupervised segmentation algorithm that automatically determines the number of clusters used for segmentation by iteratively merging and splitting clusters based on intra- and inter-cluster dispersion terms. These terms—that include the minimum number of voxels within a cluster (Φ_N_), the minimum inter-cluster distance (Φ_C_), and the maximum allowed intra-cluster dispersion (Φ_S_)—can be set prior to and may allow for certain control over the segmentation process. For this study values for Φ_C_ were derived by calculating the Mahalanobis distance between contra-lesional white matter (WM) and grey matter (GM) [2, 14]. Φ_S_ was obtained by calculating the standard deviation of WM values over all datasets within each brain. An initial guess of the number of clusters (k) was necessary to initialize the algorithm. The initial *k* cluster means were calculated using a semi-randomized approach where the new cluster mean (*µ*_*j*_) was chosen to be proportional to a randomly selected fraction of the total distance to all the previously selected cluster means [35]. K defined the cluster range in which splitting and merging was allowed and was defined as 0.5*K < K < (2*K + 1). At each iterative step, voxels were assigned to the clusters where the Mahalonobis distance to the centroid (i.e. cluster mean) was smallest. Clusters with less than Φ_N_ voxels were discarded and their voxels were redistributed over the remaining clusters based on their smallest relative distance. Subsequently, the intra-cluster distances (D_intra_: the average Mahalanobis distance between voxel vectors and the cluster centroid) and inter-cluster distances (D_inter_: the Mahalanobis distance between two cluster centroids (*µ*_*i*_*, µ*_*j*_)) were calculated. When D_intra_ was more than Φ_S_ or D_inter_ was less than Φ_C_, clusters were split or merged respectively. Subsequently, the cluster means were recalculated and a new iteration was initiated provided the stopping criteria had not been met. Clustering was completed when the algorithm converged or a maximum number of iterations (*I*) was reached. The stopping and merging criteria were based on maximum number of iterations (*I*), convergence error threshold (ɛ_r_), and number of merging steps per iteration (L). For this study Φ_N_,k,K *I*, ɛ_r_, and L were empirically derived and set to: 100, 6, 8, 100, 0.0001, and 1, respectively, to ensure convergence of the algorithm.

Additional spatial contiguity constraints were previously suggested to reduce the influence of noise and outliers on the ISODATA clustering results [2]. In this study, ISODATA was extended with spatial contiguity constraints that for each voxel calculated the local neighborhood intensity homogeneity weighted by the distance of the neighboring voxels to the voxel in the center prior to ISODATA clustering [36]. For each animal’s MRI dataset, a *t* x *f* feature matrix was created for each voxel (*v*)—for which *t* represents the number of time points and *f* the number of features per time point—and evaluated using ISODATA. To further reduce temporal artifacts particularly at tissue boundaries, the resultant ISODATA maps were pruned according to the amount of temporal dispersion measured using coefficient of variance (CoV) analysis. CoV was employed to reduce the influence of small noisy fluctuations in MRI indices over time. Temporal cluster dispersion was measured as the ratio between its temporal standard deviation and its mean. A cluster with dispersion above a pre-defined threshold was considered abnormal; clusters with less dispersion were merged into the normal tissue cluster.

Because the number of clusters identified may depend on the data used for clustering and the order in which the cluster values are assigned by ISODATA may vary per segmentation, the pruned maps were normalized using previously described techniques [16]. Pruned maps were scaled by assigning the cluster matching the cerebrospinal fluid (CSF) region to 100 and the cluster matching the contralateral WM region to 1, the remaining clusters identified by the algorithm were scaled to values between 1 and 100 depending on the difference of each cluster’s mean centroid and those of the CSF or contralateral WM regions [16].

For group comparisons, global group evolution profiles or signatures were obtained by binning in groups of 10 the normalized signatures within the ipsilateral hemisphere. Values below signature 5 were classified as a signature consistent with “normal”. Remaining signatures were binned in steps of 10 and represented regions of abnormal tissue signatures.

### Statistical analysis

**Fig. 7:**
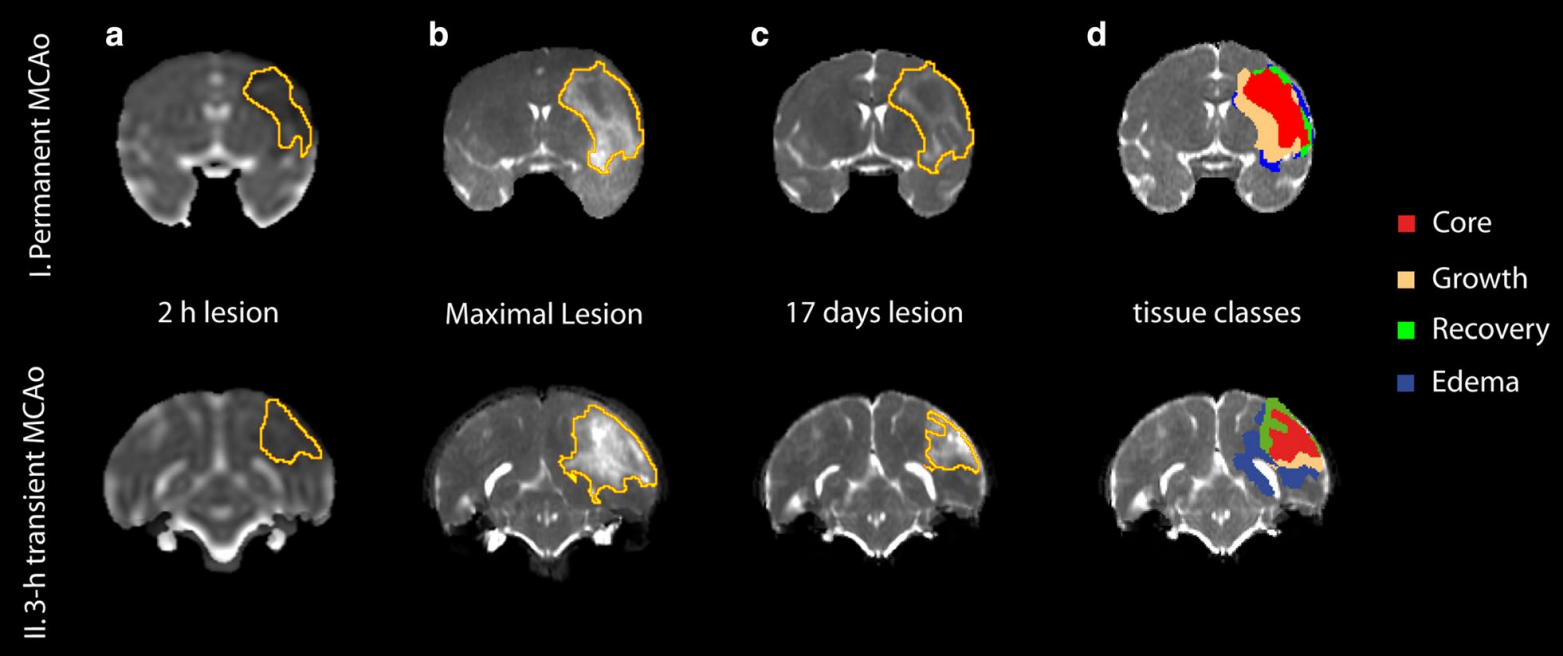
Evolution of tissue lesion volumes over time. Temporal-evolution maps (coronal view) from the brains of two macaques that underwent (I) permanent or (II) 3 h transient MCAo. Regions-of-interests of ADC-derived lesion volume at 2 h (I.**a** and II.**a**); T_2_-derived lesion at 72 (I.**b**) or 144 h (II.**b**); and T_2_-derived lesion at 17-day follow-up (I.**c** and II.**c**) were manually outlined. Voxels that were abnormal acutely (**a**) and at follow-up (**c**) were operationally defined as *Core* (**d** *red*); voxels abnormal at follow-up (**c**), but normal at the acute stage (**a**) were considered *Growth* areas (**d**: *copper*); voxels abnormal in the acute stage (**a**), but normal at 17 days follow-up (**c**) represented *Recovery* regions (**d**.I: *green*); and voxels transiently abnormal at the time-point with maximum lesion volume (**b**), but normal at acute (**a**) and chronic (**c**) stages were considered areas of *Edema* (**d**: *blue*)

Region-of-interests (ROIs) were manually outlined by two experienced researchers blinded to the ISODATA results. These manually delineated lesion volumes were enlarged with three 3-dimensional dilation steps. Four tissue classes were operationally defined as: ‘*Core*’, ‘*Growth*’, ‘*Recovery*’, or ‘*Edema*’. Voxels within the 1-h ADC lesion that overlapped with the “chronic” lesion outlined on the 17-day T_2_ map were used to define ‘*Core*’ voxels. Voxels that did not overlap were subdivided in voxels that were within the “chronic” lesion, but not within the ‘Core’. These were defined as lesion *Growth* voxels. Voxels included in the ‘Core’, but not in the “chronic” lesion, were marked as lesion *Recovery* regions. Areas of *Edema* were defined as voxels that appeared abnormal at the time-point for which the maximum T_2_ lesion volume occurred (‘*Maximal Lesion*’), but were normal at both the 1-h and 17-day time points (Fig. 7). All remaining voxels were classified as normal tissue.

Five combinations of input MRI-parameters were considered: ADC, FA, T_2_, ADC + FA, ADC + T_2_, and ADC + FA + T_2_. Additionally, CoV thresholds were varied from 0.1 to 0.01 with steps of 0.01 to determine the threshold that resulted in highest overlap between calculated abnormal ST-ISODATA regions and the ‘*Maximal Lesion’* ROI. Overlap was calculated by subdividing the clustered voxels in voxels that were correctly classified as abnormal (TP); incorrectly classified as abnormal (FP); incorrectly as classified normal (FN); or correctly classified as normal (TN) and expressed as Dice’s similarity index (DSI = (2*TP)/(2*TP + FP + FN)) [37]. DSI of 1 represented excellent overlap, DSI of 0 no overlap.

Repeated measures one-way analysis of variance (ANOVA) with post hoc Tukey correction was used to compare DSI of the ST-ISODATA models using the different MRI input parameters or CoV thresholds. Tissue class volumes were compared using Chi Square test. Repeated-measures ANOVA with post hoc Tukey correction was used to evaluate differences between temporal signatures. Values are reported as mean ± SD. P < 0.05 was considered statistically significant.

### Histological brain tissue preparation and analysis

Animals were euthanized by an intravenous overdose of sodium pentobarbital at 30 days post-stroke induction with the exception of one animal that was sacrificed earlier (17 days) due to poor prognosis. Brains were removed and fixed with 10 % formalin followed by gross sectioning of the brains into 2.5 mm blocks, to match the MRI slice thickness and orientation. Coronal blocks were embedded in paraffin and consecutively sectioned into 6 µm thick slices from the cut face throughout the entire lesion area. Successive slices were stained with hematoxylin and eosin (H&E), myelin basic protein (MBP), and luxol fast blue (LFB), scanned (Epson^®^ Perfection 3170 Photo Scanner Epson America, Miami FL, USA), and pictures digitally stored. A template of the brain showing the boundaries of the affected regions and the outline of the brain was manually traced on the pictured sections. The stained sections were examined and rated by an experienced primate neuropathologist. Histopathological tissue features within abnormal MRI tissue signatures were assessed.

## Abbreviations

ADC: apparent diffusion coefficient
CoV: coefficient of variance
FA: fractional anisotropy
DWI: diffusion weighted imaging
DSI: Dice’s similarity index
DTI: diffusion tensor imaging
H&E: hematoxylin and eosin
ISODATA: iterative self organizing data analysis
LFB: luxol fast blue
MBP: myelin basic protein
MCA: middle cerebral artery
MCAo: middle cerebral artery occlusion
ST-ISODATA: spatially and temporally adjusted ISODATA
